# SARS-CoV-2 RNA polymerase as target for antiviral therapy

**DOI:** 10.1186/s12967-020-02355-3

**Published:** 2020-05-05

**Authors:** Luigi Buonaguro, Maria Tagliamonte, Maria Lina Tornesello, Franco M. Buonaguro

**Affiliations:** 1 Innovative Immunological Models, Istituto Nazionale per lo Studio e la Cura dei Tumori, “Fondazione Pascale”-IRCCS, Via Mariano Semmola, 52, 80131 Naples, Italy; 2 Laboratory of Molecular Biology and Viral Oncology, Istituto Nazionale per lo Studio e la Cura dei Tumori, “Fondazione Pascale”-IRCCS, 80131 Naples, Italy

## Abstract

A new human coronavirus named SARS-CoV-2 was identified in several cases of acute respiratory syndrome in Wuhan, China in December 2019. On March 11 2020, WHO declared the SARS-CoV-2 infection to be a pandemic, based on the involvement of 169 nations. Specific drugs for SARS-CoV-2 are obviously not available. Currently, drugs originally developed for other viruses or parasites are currently in clinical trials based on empiric data. In the quest of an effective antiviral drug, the most specific target for an RNA virus is the RNA-dependent RNA-polymerase (RdRp) which shows significant differences between positive-sense and negative-sense RNA viruses. An accurate evaluation of RdRps from different viruses may guide the development of new drugs or the repositioning of already approved antiviral drugs as treatment of SARS-CoV-2. This can accelerate the containment of the SARS-CoV-2 pandemic and, hopefully, of future pandemics due to other emerging zoonotic RNA viruses.

SARS-CoV-2 is a new human coronavirus identified in patients with acute respiratory syndrome in Wuhan, China in December 2019 [1, 2]. Since then, the SARS-CoV-2 infection has become a pandemic, reaching almost every Country in all Continents with more than 3 million positive and 217.000 deaths, globally (on 29/04/2020 https://gisanddata.maps.arcgis.com/apps/opsdashboard/index.html#/bda7594740fd40299423467b48e9ecf6).

Similarly to the two new human CoV emerged in the past 20 years, namely the severe acute respiratory syndrome CoV (SARS-CoV) and the Middle East respiratory syndrome CoV (MERS-CoV) [3], also the current SARS-CoV-2 is suggested to be originated as a zoonosis from bats [4]. Moreover, evidences show that in bats there is a continuous circulation of additional SARS-like and MERS-like coronaviruses able to replicate efficiently in primary human lung cells [5]. It is therefore predictable that new epidemics/pandemics due to zoonotic coronaviruses will emerge in humans in the future. Consequently, together with a preventive vaccine, effective antivirals are needed to be developed within a broader program of Global preparedness.

Inter-human transmission of coronaviruses is mediated by saliva droplets reaching the new host through coughing and sneezing, both in symptomatic and asymptomatic positive subjects [6, 7]. Moreover, active virus replication in the upper respiratory tract is observed in patients also after the peak of respiratory symptoms, which may result in prolonged viral spreading of the infection [8]. Therefore, reducing the viral titer represents a major goal in order to slow/block the disease progression as well as to significantly limit the viral shedding. The latter objective would result in the lowering of R0 from 2.5 (2.5 people infected by a positive subject) to a value < 1 (less than one person infected by a positive subject). In this way the spread of the virus would be drastically contained and lastly blocked. Finally, the reduced viral replication would give the opportunity to the adaptive humoral immune system (with the production of antibodies, hopefully neutralizing) enough time to mount a sufficient primary response capable of containing further viral replication and eradicating the infection.

SARS-CoV-2 is a positive-sense RNA virus belonging to the Orthocoronavirinae (coronavirus, CoV) family and, in particular, to the genus beta (group 2) together with the other two new human coronaviruses SARS-CoV and MERS-CoV. Similarly to all RNA viruses, the viral genome replication and transcription processes of SARS-CoV-2 depend on an RNA-dependent RNA polymerase (RdRp), which is encoded by the RNA virus to catalyze the RNA synthesis from RNA templates. Consequently, RdRp is the key enzyme in the viral biological cycle of all RNA viruses, regardless the polarity of the viral RNA genome [9, 10]. Although different for each RNA viruses, all viral RdRps are characterized by a 500–600 residue catalytic module with palm, fingers, and thumb domains forming an encircled human right hand architecture. Seven catalytic motifs are located in the RdRp palm and fingers domains, comprising the most conserved parts of the RdRp and are responsible for the RNA-only specificity in catalysis. Nevertheless, RdRps from positive-sense and negative-sense RNA viruses show differences with significant implications in the enzymatic mode of action [11]. In particular, the palm catalytic subdomain (Motif C) is the most conserved region of all monomeric viral RNA polymerases with two aspartic acid residues that coordinate the two metal ions necessary for the phosphoryl transfer reaction [12, 13]. However, while positive-sense RNA viruses show a glycine preceding the two aspartates, negative-sense RNA viruses show a serine, resulting in the alternative interactions with the metal ions [14]. Mutation of the first aspartic residue results in a complete loss of RNA polymerase activity, whereas mutations of the second aspartate diminish the polymerase activity or modify the metal cofactor requirements, but do not inactivate the enzyme [15, 16]. Negative-sense RNA viruses, have an asparagine instead of the second aspartic acid in Motif C which has been shown to enable viral RNA polymerases to use manganese (Mn^2+^) instead of magnesium (Mg^2+^) as cofactor [17].

The alignment of RdRp sequences from the three epidemic/pandemic coronaviruses, confirms the high homology and conservation along the sequence (Fig. 1a). Such homology and conservation is strongly retained when the analysis includes other positive-sense RNA virus, namely HCV, Dengue, West Nile, Zika and Yellow Fever viruses (Fig. 1b). In particular, HCV shows the greatest number of identical residues with the coronaviruses. In Motif C, the DDxVV pattern has a 100% conservation but the three coronavirus are the only ones to show a serine instead of a glycine preceding the two aspartate residues.

**Fig. 1:**
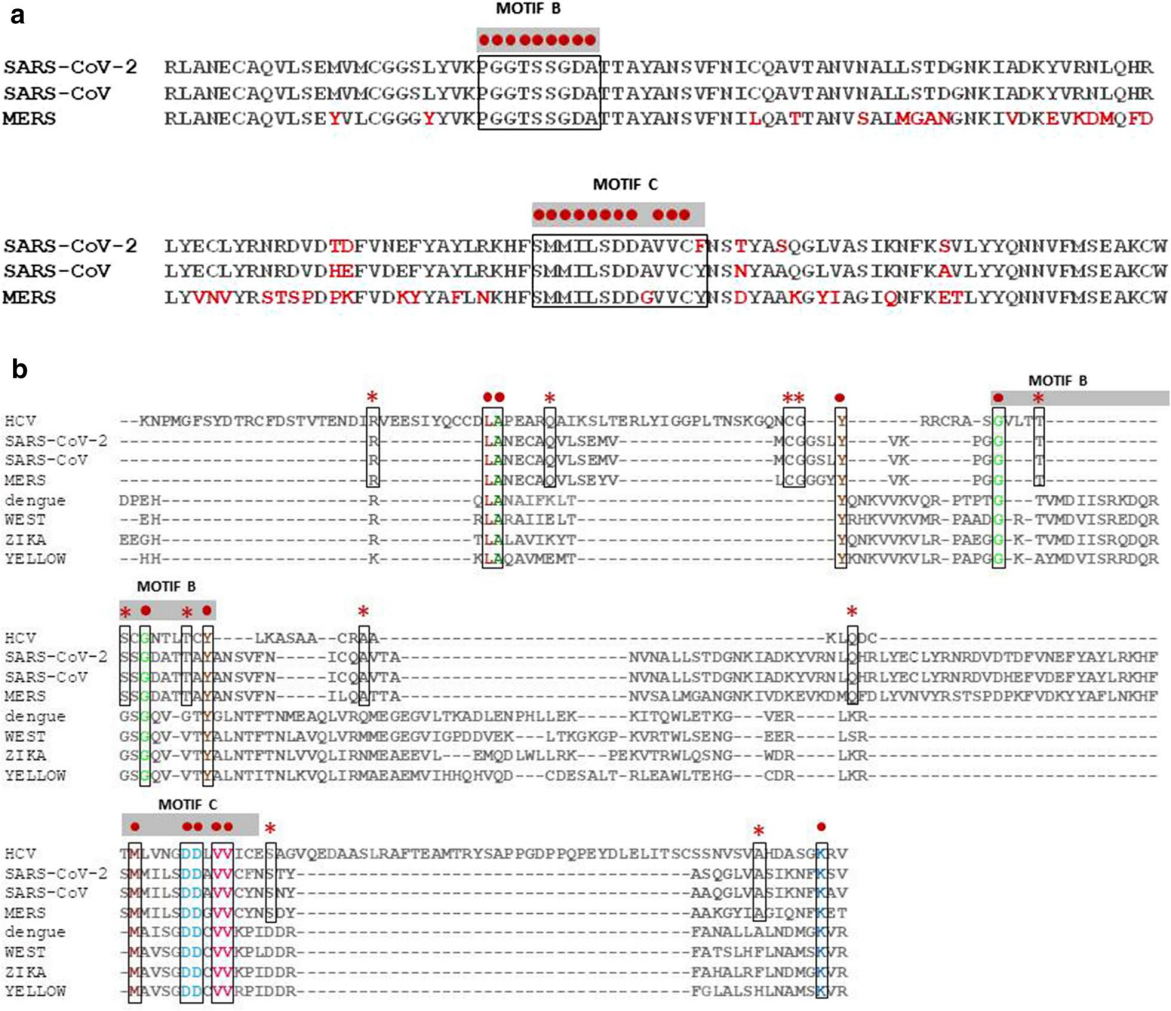

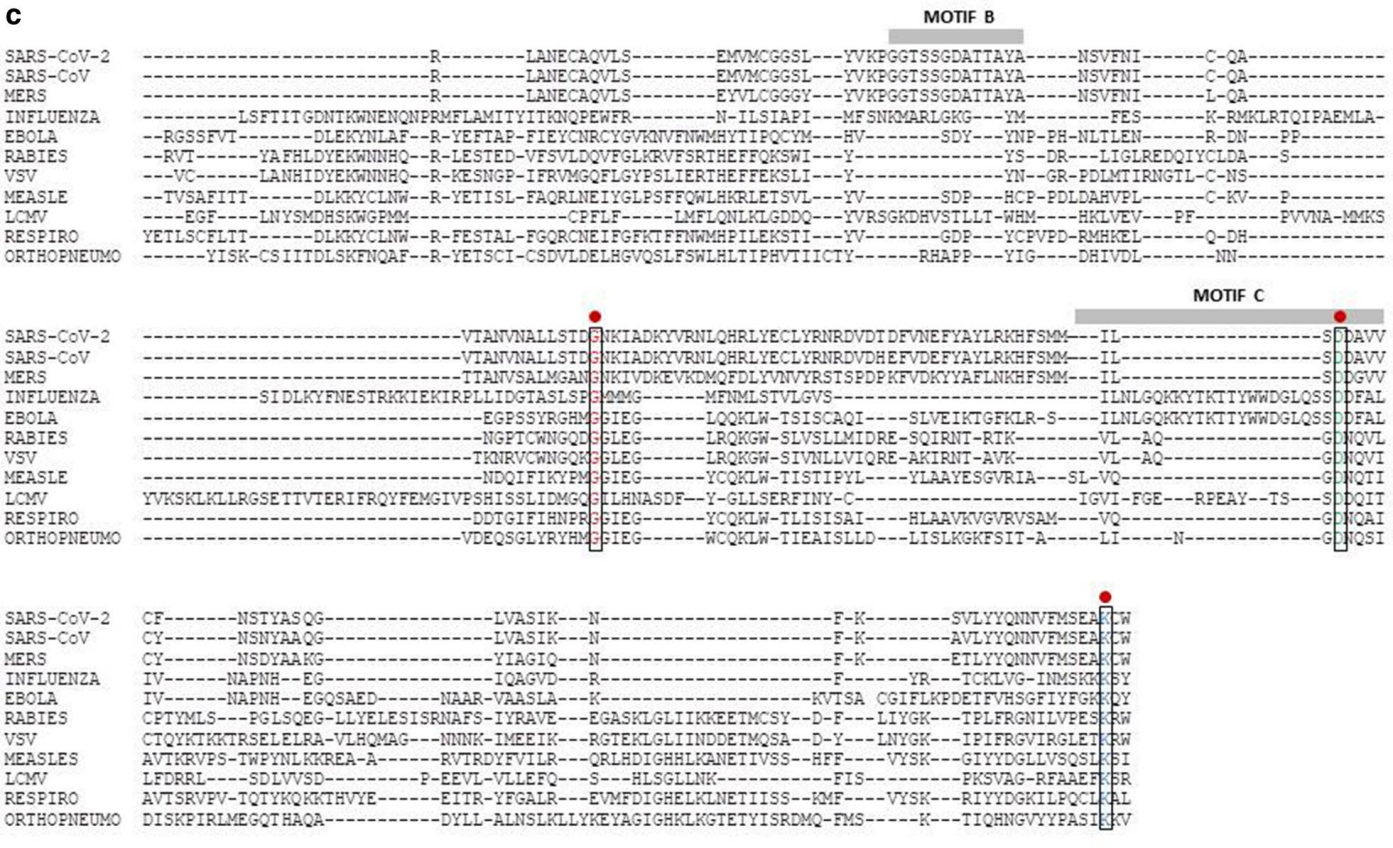
Alignment of amino acid sequences from RdRp of RNA viruses. **a** Sequences from positive-sense SARS-CoV-2, SARS-CoV and MERS viruses; **b** Sequences as in (**a**) with the addition of positive-sense viruses HCV, Dengue, West Nile, Zika and Yellow Fever viruses; **c** Sequences from positive-sense coronaviruses and negative-sense viruses Influenza, Ebola, Rabies, Vesicular Stomatitis virus, Measle, LCMV, Respirovirus and Orthopneumovirus. Red dots indicate 100% conservation of the indicated aa residues. Red asterisks indicate 100% conservation among HCV and human coronaviruses. Motif B and C of the RdRp are indicated

On the contrary, such homology is very poor when RdRp sequences from the three epidemic/pandemic coronaviruses are aligned with negative-sense RNA viruses, namely Ebola, Influenza, Rabies, Vesicular Stomatitis virus, Measle, LCMV, Respirovirus and Orthopneumovirus (Fig. 1c). As predicted, the DDxVV pattern in Motif C is not present in the negative-sense RNA viruses. The Influenza virus is the only one showing the two aspartates (DDxYY), while the other three show an asparagine substituting the second aspartate (DNxYY), suggesting the use manganese (Mn^2+^) instead of magnesium (Mg^2+^) as cofactor.

The structure modeling confirms the differences between the RdRps from positive-sense (HCV and SARS-CoV-2) and negative-sense (i.e. Influenza) RNA viruses. Indeed, considering the Motif C β-strand structure, only the alignment of RdRp structures from the two positive-sense RNA viruses results in the superimposition of the two Motifs (Fig. 2a). Furthermore, the highly conserved residues reside all in the inner part of the molecules which are in direct contact with the target genomic viral RNA and are responsible of the elongation of the nascent RNA molecule (Fig. 2b).

**Fig. 2:**
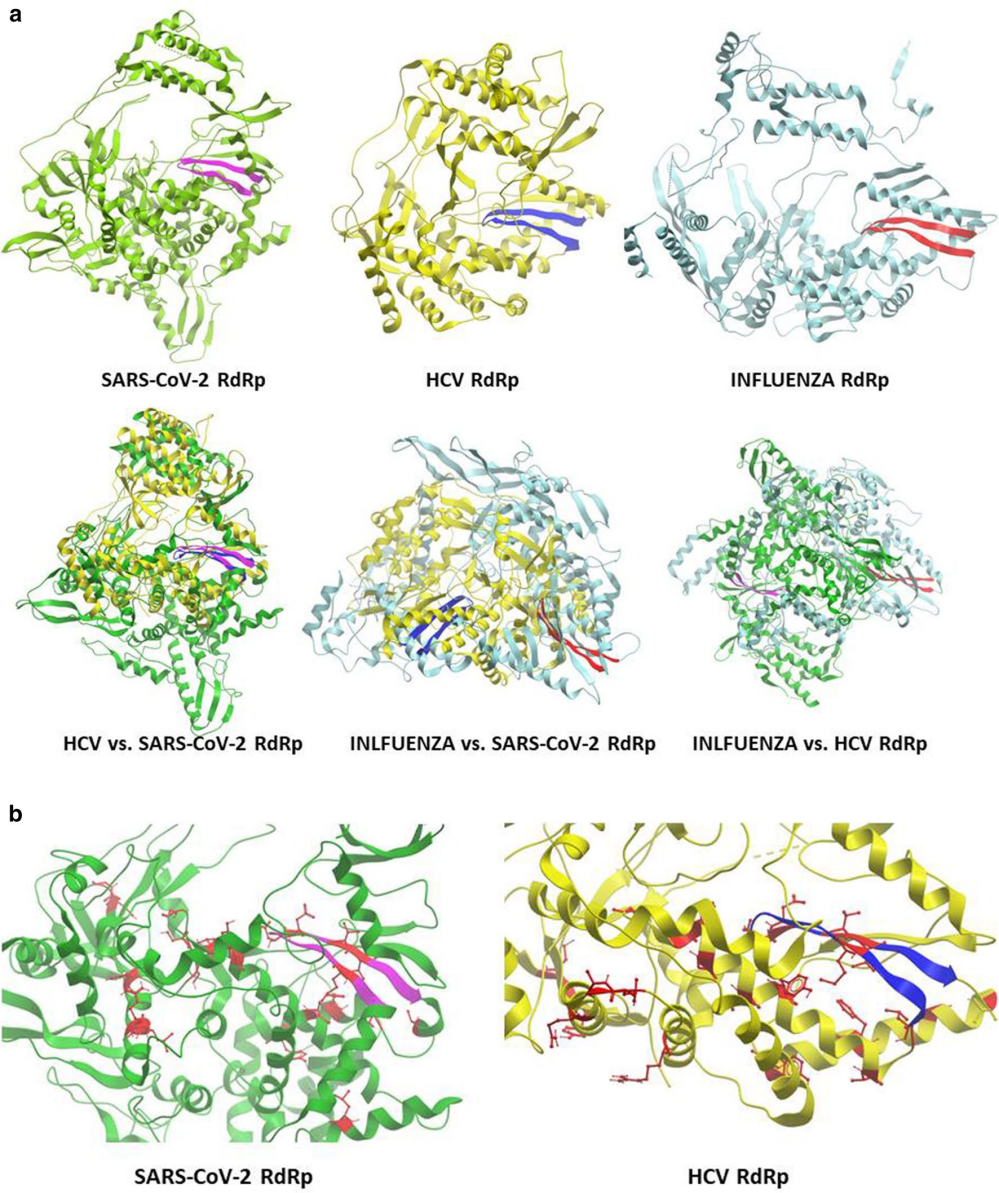
Structure modelling of the RdRps. **a** RdRp structures were derived from PDB databank: SARS-CoV-2 (6M71), Influenza virus (6QCT); HCV (3MWV). The whole molecules are presented independently or superimposed. **b** Zoomin of the SARS-CoV-2 and HCV core molecules highlighting in red color the conserved residues. Modelling was performed with Molsoft Browser

The molecular modelling which includes also the surface of molecules is a clearer representation of the overall structural difference between RdRps of positive-sense and negative-sense RNA viruses. In particular, this is clearly evidenced looking at the conformation of the core channel in which the Motif C β-strand-loop-β-strand structure protrudes (Fig. 3a–d).

**Fig. 3:**
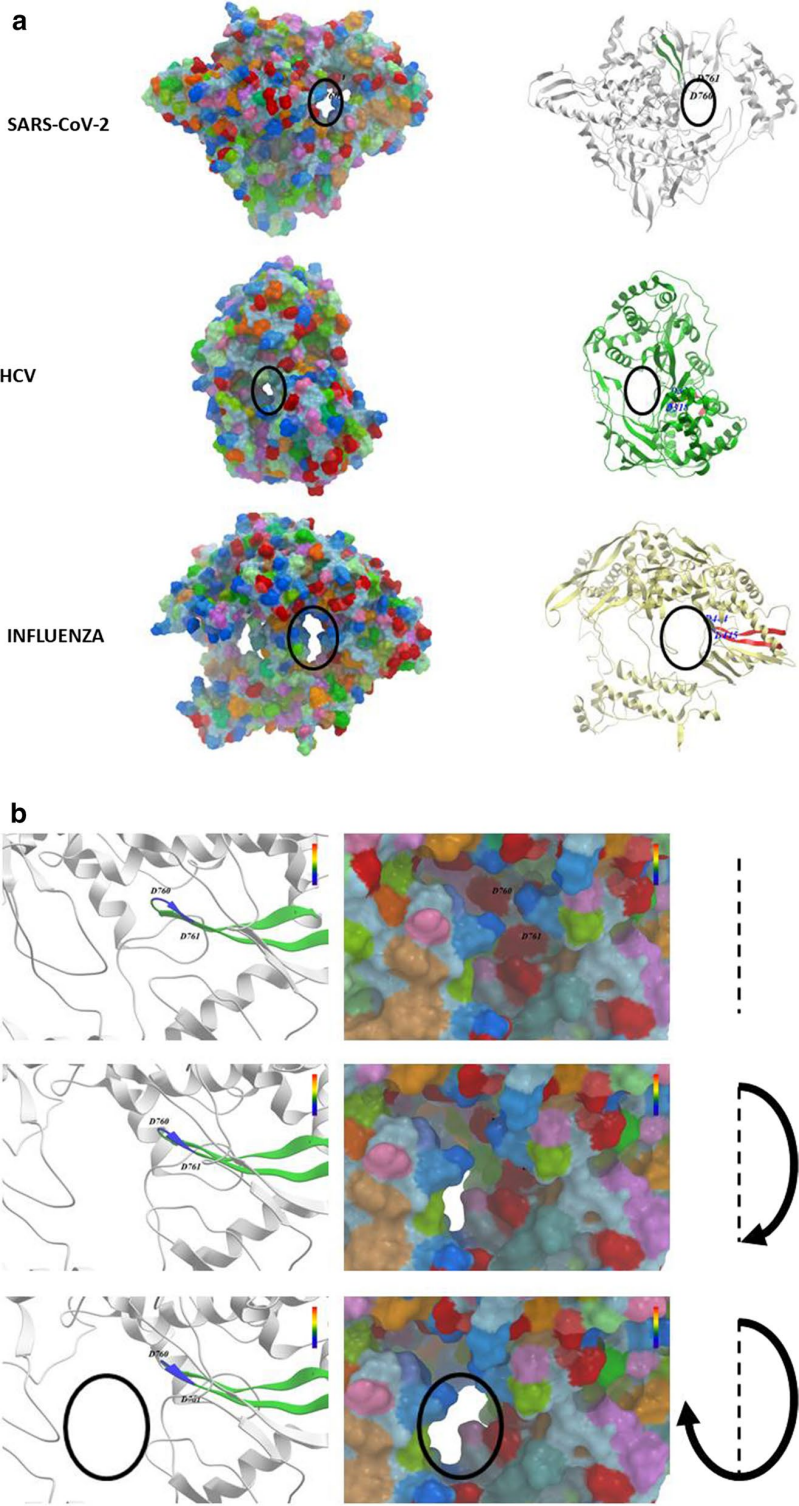

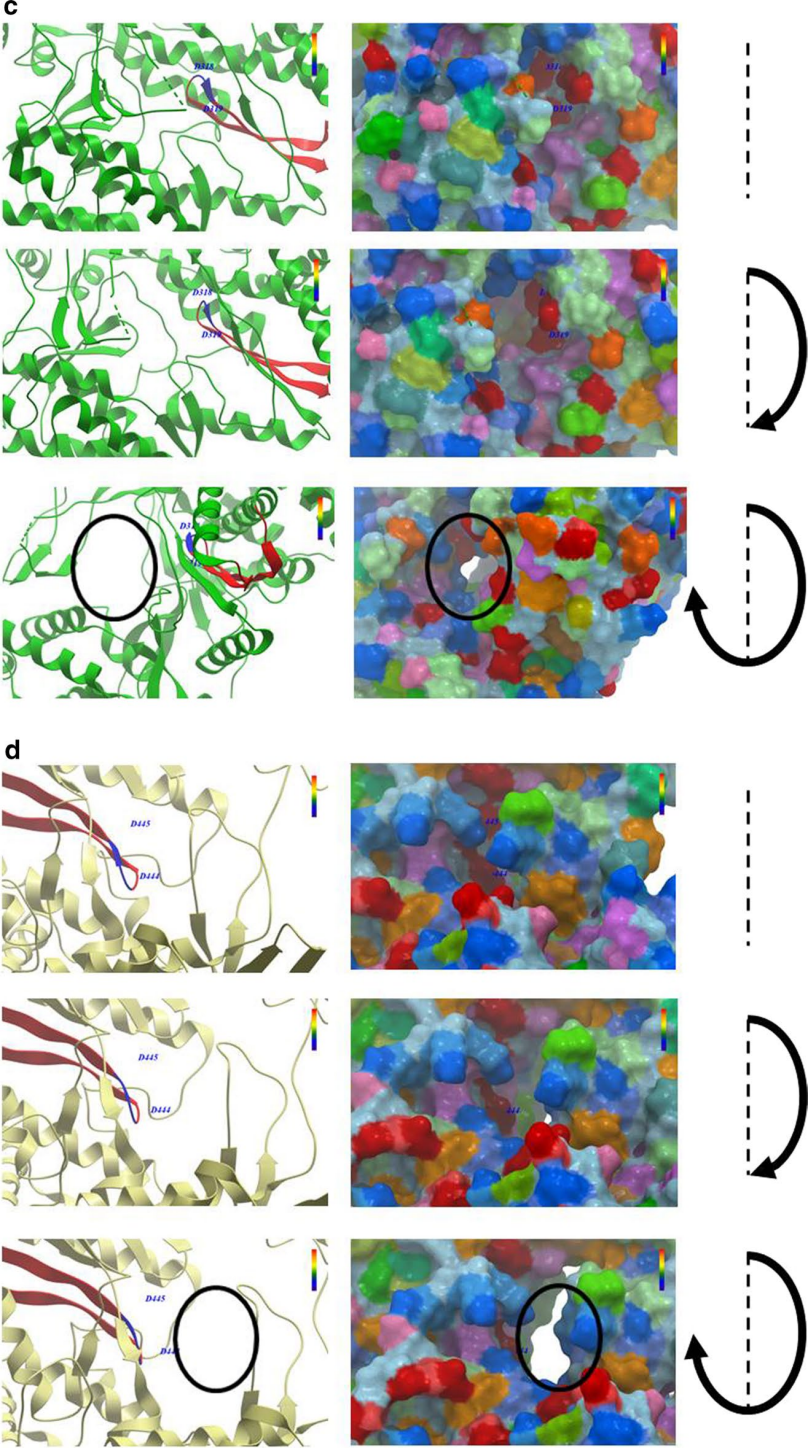
Structure modelling of the RdRps. RdRp structures were derived from PDB databank and modelling was performed as described in Fig. 2. **a** External surface and internal structures of RdRps were compared. **b** SARS-CoV-2 RdRp; **c** HCV RdRp; **d** Influenza RdRp, zoomingin the channel in which the Motif C protrudes (black empty circle). Each of the latter three panels shows three different snapshots in a clockwise rotation

Specific drugs for SARS-CoV-2 should be developed to target the RdRp regions directly involved in the viral genome replication and transcription. Nucleoside analogs would be the most obvious class of drugs to be repositioned or de novo developed. In particular, few analogs are already available originally developed to target RdRps of other RNA viruses, such as Remdesivir (Ebola virus) [18], Favipiravir (Influenza virus) [19], NHC EIDD-1931 (broad spectrum) [20] and Sofosbuvir (Hepatitis C virus) [21]. Remdesivir and Favipiravir are currently evaluated in clinical trials to assess the efficacy in SARS-CoV-2 infected subjects (Remdesivir: NCT04292899; NCT04257656) (Favipiravir: NCT04310228). NHC EIDD-1931 has been shown to inhibit SARS-CoV-2 replication in vitro and in a pre-clinical animal model [22]. However, all three of them have been developed for negative-sense RNA viruses which show a significant difference in the RdRp sequence and structure compared to the positive-sense SARS-CoV-2 RNA virus. In this respect, Sofosbuvir could represent the optimal nucleoside analog to be repositioned to treatment of SARS-CoV-2 infection. Indeed, it has been developed for the positive-sense HCV RNA virus which shares high sequence and structural homology with SARS-CoV-2. Moreover, Sofosbuvir has been already shown to be effective for other positive-sense RNA viruses, namely Yellow Fever and Hepatitis A virus [23, 24].

In conclusion, as for all RNA viruses, the RdRp of the newly identified positive-sense human SARS-CoV-2 RNA virus represents the most optimal target for an antiviral drug. Linear amino acid sequence as well as molecule structure show the highest homology to RdRps of other positive-sense RNA viruses. Therefore, it is highly predictable that an antiviral developed for an RNA virus with a genome of the same polarity (i.e. Sofosbuvir for HCV) could have a higher inhibitory efficacy against the SARS-CoV-2, compared to those developed for negative-sense RNA viruses. Overall, the possibility of repositioning already available drugs will significantly accelerate the containment of the SARS-CoV-2 pandemic and, hopefully, of future pandemics due to other emerging zoonotic RNA viruses.

## Data Availability

Not applicable.
